# Impaired Knee Joint Position Sense in Chronic Patellar Tendinopathy Is Associated with Kinesiophobia but Not Central Sensitization

**DOI:** 10.3390/jcm15103988

**Published:** 2026-05-21

**Authors:** Özlem Yener, Altınay Göksel Karatepe

**Affiliations:** 1 Department of Sports Medicine, Faculty of Medicine, Dokuz Eylül University, 35340 Izmir, Turkey; 2 Department of Physical Medicine and Rehabilitation, İzmir City Hospital, Sağlık Bilimleri University, 35540 Izmir, Turkey

**Keywords:** patellar tendinopathy, proprioception, joint position sense, kinesiophobia, central sensitization, knee, sensorimotor function

## Abstract

**Background:** Patellar tendinopathy is a common musculoskeletal condition that may impair functional performance and limit physical activity. While structural and mechanical factors have been widely investigated, the role of proprioceptive function and its interaction with behavioral and central pain-related mechanisms remain unclear. This study aimed to investigate the relationship between knee joint position sense, kinesiophobia, and central sensitization in individuals with chronic patellar tendinopathy. **Methods:** A total of 42 recreational athletes with clinically diagnosed chronic patellar tendinopathy were included in this cross-sectional study. Knee joint proprioception was assessed using joint position sense testing at multiple knee flexion angles with a continuous passive motion device. Kinesiophobia and central sensitization were evaluated using the Tampa Scale of Kinesiophobia and the Central Sensitization Inventory, respectively. Joint position sense values of the involved and contralateral extremities were compared, and correlation analyses were performed to examine associations between joint position sense and psychosocial variables. **Results:** The involved extremity demonstrated significantly greater absolute angular error compared to the contralateral side at higher knee flexion angles (≥60°, *p* < 0.01), whereas no significant differences were observed at lower angles. A moderate positive correlation was found between joint position sense error and kinesiophobia at higher flexion angles (≥30°, *p* < 0.01). No significant association was identified between joint position sense error and central sensitization across any of the tested angles (*p* > 0.05). **Conclusions:** Proprioceptive function is impaired in individuals with chronic patellar tendinopathy, particularly under increased mechanical demand. The association between joint position sense deficits and kinesiophobia, but not central sensitization, suggests a potential relationship between movement-related fear and sensorimotor alterations. These findings highlight the importance of incorporating proprioceptive assessment and addressing kinesiophobia in the clinical management of patellar 36 tendinopathy.

## 1. Introduction

Patellar tendinopathy is the process of degeneration of the patellar tendon due to repetitive explosive vertical loading among both recreational and elite athletes, and manifests as activity-associated knee pain [1,2,3]. Degeneration refers to fibrotic and disorganized tendon tissue, which leads to biomechanical changes [2]. When symptoms persist for three months or longer, the condition is classified as chronic patellar tendinopathy (CPT) [4], which can significantly impair sports participation and often requires prolonged rehabilitation approaches, including exercise-based interventions and load management strategies [1,5].

CPT, while primarily recognized for its impact on muscle strength and flexibility, may also compromise proprioceptive function, although this relationship remains underexplored [2]. Proprioception—defined as the perception of joint position, movement, and resistance without visual cues—is a key factor in maintaining functional joint stability and neuromuscular control [1,6]. Evidence suggests that proprioceptive deficits, such as impaired joint position sense (JPS) or reduced capacity for force discrimination, may occur in individuals with knee pathologies, including CPT, potentially increasing the risk of reinjury and postural instability [3,7]. Nevertheless, few studies have directly examined proprioceptive loss in the context of CPT, highlighting a gap in the current understanding of how sensory feedback mechanisms are affected in this condition [1,2].

Kinesiophobia and central sensitization (CS) are increasingly recognized as interacting mechanisms that may contribute to the chronicity and complexity of musculoskeletal conditions such as CPT. Kinesiophobia, defined as an excessive fear of movement due to anticipated pain or reinjury, can lead to reduced physical activity, altered movement patterns, and delayed functional recovery [8,9,10]. When pain persists, even if in the absence of ongoing tissue damage, sustained nociceptive input from the tendon may induce maladaptive neuroplastic changes within the central nervous system, giving rise to CS—a state of heightened neuronal responsiveness characterized by increased pain sensitivity and lowered thresholds to stimuli [11]. In athletes with CPT, prolonged mechanical loading and inflammation of the patellar tendon may serve as peripheral drivers of this sensitization, potentially maintaining pain even after structural healing has occurred [6,11]. Importantly, fear of movement and avoidance behaviors associated with kinesiophobia may further perpetuate CS by limiting neuromuscular engagement and reinforcing aberrant pain processing circuits [9,11]. Although direct empirical evidence linking CS and kinesiophobia to CPT remains scarce, the observed overlap in their behavioral and neurophysiological features suggests a plausible bidirectional relationship that may underlie persistent symptoms. Investigating these dimensions within the context of CPT could provide valuable insights into more comprehensive, biopsychosocial models of treatment and rehabilitation.

To the best of our knowledge, no previous study has investigated the combined effects of kinesiophobia and CS on JPS in individuals with CPT. Given the proposed interplay between psychological and neurophysiological factors in the persistence of pain and functional limitation, examining their association with proprioceptive function may provide crucial insights into the underlying mechanisms of CPT. While current diagnostic and therapeutic frameworks have largely focused on structural tendon abnormalities, this reductionist view may fail to address the complex sensorimotor alterations influenced by fear of movement and heightened central pain processing. Therefore, this study aims to explore the relationship between kinesiophobia, CS and JPS—a core component of proprioception—in recreational athletes with CPT. By investigating these novel associations, the present research seeks to expand the existing biopsychosocial understanding of tendinopathy and support the development of more individualized and comprehensive rehabilitation approaches.

## 2. Methods

### 2.1. Study Design

This cross-sectional observational research explored the relationship between kinesiophobia, CS, and JPS in recreational athletes with CPT. Data collection was carried out between August 2024 and January 2025 at the Departments of Sports Medicine with Physical Medicine and Rehabilitation of Sağlık Bilimleri University İzmir City Hospital, under standardized laboratory conditions to ensure consistency in testing procedures.

### 2.2. Participants

Based on an a priori power analysis conducted using G*Power (version 3.1.9.7, Kiel University, Kiel, Germany), a total sample size of 42 participants was determined to be required for the study, considering a 95% confidence level (1–α), 95% statistical power (1–β), and a medium effect size (d = 0.5) for a two-tailed point-biserial correlation analysis. Thus, 42 recreational athletes clinically diagnosed with unilateral CPT by an experienced specialist in sports medicine (OY) participated in this study. Participants were recruited through referrals and direct admissions to the sports medicine outpatient clinic. Inclusion criteria were (1) age between 18 and 50 years, (2) engaging in sports activities for a total duration of at least 90 min per week, (3) experiencing pain localized at the patellar tendon during sports activity for at least three months, (4) clinical confirmation of unilateral CPT, and (5) a Victorian Institute of Sport Assessment–Patella (VISA-P) score of ≤80. Exclusion criteria included (1) history of lower-extremity surgery; (2) concurrent lower-extremity musculoskeletal injuries; (3) any neurological, vestibular, visual and/or metabolic disorders affecting sensorimotor function; (4) any cognitive and/or mental disorder; and (5) use of pain-modulating medication within 48 h prior to assessment. The recorded demographic variables included age, gender, height, weight, body mass index (BMI), education level, sports experience, type of sport, training volume, sports participation level and dominant leg side. Information regarding the interest in sports was determined through the self-reported verbal responses of participants. The leg used preferentially for kicking a ball was considered the dominant leg.

### 2.3. Clinical Assessments

Each participant was clinically diagnosed with unilateral CPT, all primarily referring to sports-related overuse rather than acute traumatic injury.

The final diagnosis was based on a comprehensive physical examination, which remains the criterion standard for identifying patellar tendinopathy in both athletic and recreational populations [12]. The hallmark clinical finding was focal point tenderness at the patellar tendon and/or its insertions upon palpation, consistent with the most prevalent diagnostic indicator. Additionally, pain mapping was performed using a single-legged squat to help localize tendon-specific pain and distinguish it from other sources of anterior knee discomfort [12]. To support the clinical diagnosis and assess symptom severity, the VISA-P questionnaire was administered. VISA-P is a widely accepted, self-administered instrument designed to evaluate the severity of symptoms and functional limitations in patients with patellar tendinopathy. It consists of eight items—six assessing pain during daily activities and two assessing sports participation ability—with total scores ranging from 0 to 100. Higher scores indicate fewer symptoms and better function [13], while scores ≤ 80 are indicative of patellar tendinopathy [3,5]. The Turkish version of VISA-P has demonstrated valid and reliable psychometric properties [14]. While imaging modalities such as ultrasonography and magnetic resonance imaging may aid in confirming patellar tendinopathy, they were not employed in this study due to practical constraints. Ultrasonography, although non-invasive, is highly operator-dependent and limited in ruling out intra-articular pathology. Magnetic resonance imaging offers better sensitivity (78%) and broader diagnostic capability but is less accessible, more costly, and time-consuming [13]. Therefore, palpatory tenderness, pain provocation during functional tasks, and VISA-P scoring were considered sufficient for participant inclusion. After determining eligibility for inclusion, clinical data regarding the involved side, duration of symptoms, and scores of the Tampa Scale of Kinesiophobia (TSK) with Central Sensitization Inventory (CSI) were collected for each participant. The TSK is a self-report instrument designed to assess fear of movement or reinjury, particularly in individuals exhibiting fear-avoidance behaviors [8]. The questionnaire includes 17 items rated on a 4-point Likert scale, ranging from 1 (strongly disagree) to 4 (strongly agree), with a total score between 17 and 68. Higher scores reflect greater levels of kinesiophobia, and a cut-off score of ≥37 is typically interpreted as indicative of clinically significant fear of movement [15]. The Turkish version of TSK has been shown to be both valid and reliable [16]. The CSI is a self-reported questionnaire developed to assess symptoms associated with CS. Part A of the inventory consists of 25 items rated on a 5-point Likert scale (0 = never to 4 = always), yielding a total score ranging from 0 to 100. Higher scores reflect greater symptom burden related to CS [11,17]. A score of ≥40 is generally considered indicative of clinically significant CS [11]. Part B documents whether the individual has received medical diagnoses commonly associated with CS [17]. Data of Part A were recorded for the analysis [18]. Evidence from validation studies indicates that the Turkish version of CSI possesses robust reliability and validity [17].

### 2.4. Measurement of JPS

Proprioceptive function of the knee joint was evaluated through the JPS test, which measures the ability to reproduce specific knee angles [19,20]. The test was conducted with an Artromot Active-K continuous passive motion device (Ormed GmbH, Freiburg, Germany) during a single measurement session at the Department of Physical Medicine and Rehabilitation. The examiner (AGK) was blinded to the side affected by CPT, and measurements were first performed on the dominant extremity, followed by the contralateral side in all participants. In the testing procedure, the participant was positioned supine, with the tested extremity aligned to the axis of the device, allowing free knee flexion and extension. In order to prevent cutaneous sensory input from clothing, all participants were evaluated while wearing shorts. Visual input was eliminated using a blindfold to avoid any visual cues during the test. The assessment was conducted at eight different knee flexion angles (0°, 15°, 30°, 45°, 60°, 75°, 90°, and 120°). The device passively moved the extremity at a constant angular velocity of 2°/s [19]. At each of the eight target angles, the joint position was first demonstrated passively and repeated five times to familiarize the participant with the target angle. During the test phase, the device again moved the extremity passively, and participants were asked to estimate the target angle during this motion and press a button on the device when they believed the correct position had been reached. The device automatically displayed the angular deviation between the target and reproduced positions. Three consecutive trials were conducted at each angle, and the mean of these three values was recorded as the absolute angular error, representing proprioceptive accuracy without directional bias [2,6,8,19]. The same procedure was repeated on the contralateral extremity to enable within-subject comparison.

### 2.5. Statistical Analyses

Descriptive statistics were reported as mean ± standard deviation for normally distributed continuous variables, and as median with interquartile range for non-normally distributed ones. Categorical variables were expressed as frequencies and percentages. The distribution of data was assessed using the Shapiro–Wilk test to determine the appropriate statistical tests. In order to compare JPS between the involved and healthy extremities, the paired samples *t*-test was used for normally distributed data; otherwise, the Wilcoxon signed-rank test was applied. The relationship between JPS values of the involved extremities and scores of TKS with CSI was explored through correlation analyses of Pearson’s or Spearman’s coefficients, depending on data distribution characteristics. Correlation strength was interpreted as follows: 0–0.19 = very weak, 0.20–0.39 = weak, 0.40–0.69 = moderate, 0.70–0.89 = strong, and 0.90–1.0 = very strong [15]. Statistical significance was set at *p* < 0.05 for all tests. All analyses were conducted using IBM SPSS (Statistical Package for the Social Sciences) Statistics for Windows version 30.0 (IBM Corp., Armonk, NY, USA).

## 3. Results

The demographic and clinical characteristics of participants are presented in Table 1. The study sample consisted of 42 recreational athletes forming a clinically defined cohort diagnosed with CPT.

Table 2 depicts the comparison of knee JPS between the involved and healthy extremities. The involved extremity demonstrated greater absolute angular error values compared to the contralateral side; however, these differences reached statistical significance only at higher knee flexion angles (≥60°; *p* < 0.05). No significant differences were observed at lower flexion angles. These findings indicate that proprioceptive deficits in recreational athletes with CPT are more pronounced at higher degrees of knee flexion, suggesting an adverse effect of increased mechanical demand on JPS accuracy.

The relationships between proprioceptive performance and psychosocial variables are shown in Table 3. A moderate positive correlation was observed between JPS error and kinesiophobia at higher knee flexion angles (≥30°; *p* < 0.05), indicating a potential association between greater fear of movement and reduced repositioning accuracy. In contrast, no significant association was found between JPS error and CS across any of the tested angles (*p* > 0.05).

## 4. Discussion

The present study investigated the relationship between knee joint proprioception, kinesiophobia, and CS in recreational athletes with CPT. The main results revealed that proprioceptive accuracy was impaired in the involved extremity, particularly at higher degrees of knee flexion, with kinesiophobia showing a moderate association with increased JPS error, whereas no significant relationship was observed with CS. These findings suggest a possible link between proprioceptive impairment and movement-related fear in recreational athletes with CPT. Nevertheless, the potential influence of factors such as sex, sport type, symptom duration, and training characteristics should also be considered when interpreting these findings. To the best of our knowledge, this is the first study to examine these variables concurrently in the context of patellar tendinopathy, providing novel insight into the interaction between sensorimotor function and pain-related factors.

### 4.1. JPS

Proprioception of the knee joint relies on the integrated input from muscle spindles, Golgi tendon organs and joint-related mechanoreceptors, each contributing differently depending on joint position, movement characteristics and loading conditions [2,6]. While muscle spindles are considered the primary contributors to JPS, particularly within mid-range movements, Golgi tendon organs are more responsive to changes in tendon tension and mechanical load [2]. Therefore, alterations in tendon structure and mechanical properties, as observed in patellar tendinopathy, may disrupt afferent signaling and lead to deficits in proprioceptive accuracy. Beyond its role in JPS, proprioception is a key determinant of neuromuscular control and postural stability, and impairments in this system may increase susceptibility to subsequent lower-extremity injuries. In patients with patellar tendinopathy, pain-related alterations have been associated with reduced dynamic postural stability, indicating a potential link between sensory deficits and impaired motor control [5]. In this context, the proprioceptive deficits observed in the present study may not only reflect a localized sensorimotor impairment but also represent a clinically relevant factor contributing to injury risk, with impairments becoming more evident at higher degrees of knee flexion.

Previous studies investigating proprioception in patients with patellar tendinopathy have reported conflicting results. While some authors suggested the presence of proprioceptive deficits or asymmetries between extremities [3], others did not observe alterations in JPS, particularly when assessments were limited to mid-range angles (e.g., 30–60° of knee flexion) or when different proprioceptive modalities, such as threshold to detect passive motion, were employed [1,2]. In addition, various studies evaluated proprioception under non-functional conditions, including low angular velocities or non-weight-bearing positions, which may not adequately reflect the sensorimotor demands encountered during dynamic and load-bearing activities [1,6]. These inconsistencies may also be related to heterogeneity in study populations, particularly regarding symptom chronicity and athletic background, as samples often included mixed groups without a clearly defined chronic presentation. In contrast, our study evaluated JPS across a wider range of motion, providing a more functionally relevant and clinically applicable perspective. From a sports medicine standpoint, these findings highlight the importance of considering proprioceptive function as a clinically meaningful component in the assessment and rehabilitation of recreational athletes with CPT.

### 4.2. Kinesiophobia

The moderate association observed between kinesiophobia and JPS error in the present study aligns with previous findings in knee osteoarthritis populations, where fear of movement has been shown to correlate with increased joint position error, pain intensity, and reduced functional performance [8]. In these populations, kinesiophobia has also been identified as a significant predictor of proprioceptive accuracy and functional outcomes, suggesting a broader role of fear-related mechanisms in sensorimotor control.

From a theoretical perspective, kinesiophobia may be related to altered proprioceptive performance through changes in motor control strategies and neuromuscular activation patterns. Previous literature has suggested that fear of movement may be linked to changes in muscle activation, increased joint stiffness, and modified afferent input, which could potentially influence JPS. Furthermore, neurophysiological evidence indicates that fear-related cognitions, including pain catastrophizing, are associated with altered central processing, particularly in brain regions involved in attention, anticipation, and emotional modulation of pain, which may indirectly affect sensorimotor integration [8].

In addition, previous evidence indicates that increased kinesiophobia is associated with worsening pain, reduced range of motion, and impaired functional performance [15], supporting the notion that fear-avoidance behaviors may be associated with a cycle of disuse, neuromuscular inefficiency, and proprioceptive decline. However, it should be noted that these findings are predominantly derived from osteoarthritis or post-surgical populations, which differ substantially from the chronic tendinopathy profile examined in the present study.

In this context, our study extends the existing literature by demonstrating that even in a relatively young and physically active population with CPT, kinesiophobia is associated with impaired proprioceptive accuracy. Unlike previous studies focusing on degenerative joint conditions, the present findings suggest a potential relationship between fear of movement and proprioceptive alterations independent of advanced structural joint pathology. This distinction may indicate that kinesiophobia is not limited to chronic degenerative conditions but may also be clinically relevant in chronic overuse-related tendon disorders such as patellar tendinopathy.

### 4.3. CS

In contrast to kinesiophobia, no significant association was observed between JPS and CS in the present study. This finding aligns with previous evidence suggesting that lower-extremity tendinopathies may predominantly reflect localized rather than widespread nociceptive processing. For instance, patients with patellar and Achilles tendinopathy have been shown to exhibit altered sensory responses at the affected tendon without corresponding changes at remote sites, indicating a lack of generalized CS features [9].

This localized pain profile may explain the absence of a relationship between CS and proprioceptive performance in our cohort. Although CS has been reported in various chronic musculoskeletal conditions, its presence in tendinopathy appears to be inconsistent and potentially dependent on factors such as symptom severity, chronicity, and population characteristics. Particularly in athletic populations, pain mechanisms may remain more peripherally driven despite chronic symptom duration.

Furthermore, evidence from studies investigating myofascial pain suggests that CS symptoms may increase without necessarily reaching clinically meaningful thresholds for central sensitivity syndromes, highlighting a distinction between symptom amplification and clinically significant central involvement [11]. This may partially account for the lack of association observed in the present study. Taken together, these findings may suggest a closer association of sensorimotor alterations in patellar tendinopathy with behavioral and perceptual factors than with widespread central nociceptive mechanisms.

From a clinical point of view, the observed impairment in JPS suggests that proprioceptive function should be considered an important parameter during both follow-up and rehabilitation in recreational athletes with CPT. Monitoring and addressing these deficits may be relevant for optimizing functional recovery. In this context, incorporating balance and proprioceptive training into rehabilitation programs may be beneficial, particularly in the presence of concomitant sensorimotor impairments.

In addition, kinesiophobia should not be overlooked in clinical practice, as it may influence movement behavior and rehabilitation outcomes. Addressing fear of movement through patient education, graded exposure to activity, and the integration of psychologically informed strategies may support more effective and sustainable recovery.

## 5. Limitations and Strengths

To the best of our knowledge, this is the first study to investigate the relationship between knee JPS, kinesiophobia, and CS in individuals with patellar tendinopathy, providing novel insight into the interplay between sensorimotor and behavioral factors in this population. Some limitations should be considered when interpreting the findings. The cross-sectional design, the absence of a healthy control group, and the inclusion of a mixed-gender sample without sex-specific analysis may limit causal interpretation and the generalizability of the results. In addition, the diagnosis of patellar tendinopathy was based on clinical evaluation without imaging confirmation. Detailed information regarding previous treatment history and recurrence characteristics was also not systematically collected. Potentially confounding factors such as sex, sport type, symptom duration, and training characteristics could not be analyzed separately due to the relatively small and heterogeneous sample. Furthermore, the greater impairment observed at higher knee flexion angles may partially reflect a fatigue-related effect of the testing protocol, which should be considered in future research designs. The absence of formal multiple comparison correction methods represents another limitation of the study, as the relatively small sample size and exploratory design restricted the feasibility of more comprehensive statistical approaches. Despite these limitations, the study has several strengths. The within-subject design may be considered a key methodological strength, as it minimizes inter-individual variability and allows for a more sensitive comparison between involved and contralateral extremities. In addition, the use of a standardized and controlled measurement protocol across multiple knee flexion angles enhances the robustness of the proprioceptive assessment. Furthermore, the simultaneous evaluation of proprioceptive function together with both kinesiophobia and CS provides a more comprehensive understanding of factors potentially influencing sensorimotor performance.

## 6. Conclusions

In recreational athletes with CPT, impaired knee JPS was potentially associated with higher levels of kinesiophobia, whereas no significant association was observed with CS. Proprioceptive function may represent an important yet underrecognized component in individuals with patellar tendinopathy. From the perspective of sports medicine, it should be considered not only as a neurosensory parameter but also as a clinically meaningful factor in both assessment and rehabilitation, with potential implications for injury risk and recovery. Nevertheless, the findings should be interpreted in the light of the correlational design and the relatively small heterogeneous sample of the study. Finally, the potential future role of kinesiophobia in the chronicity of patellar tendinopathy remains to be studied.

## Figures and Tables

**Table 1:** Demographic and clinical variables of the participants.

Age, years	26.7 ± 7.4
Gender	
Female	17 (40%)
Male	25 (60%)
Height (m)	1.73 ± 0.26
Weight (kg)	71.48 ± 11.89
BMI (kg/m^2^)	22.39 ± 2.26
Education level	
High school	9 (21%)
University	33 (79%)
Sports experience (y)	6.5 (6)
Type of sport	
Basketball	16 (38%)
Volleyball	11 (26%)
Soccer	9 (21%)
Crossfit	4 (10%)
Tennis	2 (5%)
Training volume (h/w)	7 (6)
Sports participation level	
Recreational	6 (14%)
Amateur competitive	36 (86%)
Dominant side	
Right	39 (93%)
Left	3 (7%)
VISA–P (score)	60.3 ± 5.9
Involved side	
Right	35 (83%)
Left	7 (17%)
Duration of symptoms (mo)	18.5 (13)
TSK (score)	50.29 ± 9.43
CSI (score)	43.90 ± 11.02

Data are expressed as mean ± standard deviation, median (interquartile range) or number (%). BMI: body mass index, CSI: Central Sensitization Inventory, h: hour, kg: kilogram, m: meter, mo: month, TSK: Tampa Scale of Kinesiophobia, VISA–P: Victorian Institute of Sport Assessment–Patella, w: week, y: year.

**Table 2:** Comparison of knee joint position sense errors between the involved (chronic patellar tendinopathy) and healthy extremities.

	Involved Extremity (°)	Healthy Extremity (°)	*p*-Value
0°	1.92 ± 2.19	1.68 ± 2.01	0.589
15°	0.99 (2.08)	1.17 (1.96)	0.102
30°	2.63 (5.16)	2.92 (4.73)	0.373
45°	2.59 (3.96)	1.77 (3.14)	0.099
60°	5.95 ± 4.65	4.57 ± 3.84	**0.041**
75°	6.47 ± 3.30	4.03 ± 2.80	**0.046**
90°	8.31 ± 5.97	6.99 ± 5.02	**0.024**
120°	5.72 ± 5.66	3.68 ± 2.93	**0.032**

Values represent absolute repositioning error (degrees). Data are expressed as mean ± standard deviation or median (interquartile range). Bold data express the statistical significance (*p* < 0.05).

**Table 3:** Correlation between the absolute angular error values of involved extremities and scores of the Tampa Scale of Kinesiophobia with Central Sensitization Inventory.

		TSK (Score)		CSI (Score)	
		r	*p*	r	*p*
Absolute angular error	0°	0.362	0.179	−0.016	0.937
	15°	0.407	0.124	0.047	0.802
	30°	0.461	0.006 *	0.103	0.553
	45°	0.512	0.002 *	0.117	0.482
	60°	0.584	<0.001 **	0.071	0.671
	75°	0.543	0.004 *	0.089	0.594
	90°	0.631	<0.001 **	0.064	0.702
	120°	0.477	0.005 *	0.108	0.503

Bold data express the statistical significance (* *p* < 0.05, ** *p* < 0.001).

## Data Availability

The datasets used and/or analyzed during the current study are available from the corresponding author on reasonable request.
